# Stereotactic Body Radiotherapy for a Sacral Metastasis Clarified by Diffusion-Weighted Whole-Body Imaging With Background Body Signal Suppression in a Patient With Castration-Resistant Prostate Cancer

**DOI:** 10.7759/cureus.23047

**Published:** 2022-03-10

**Authors:** Atsuto Katano, Kenta Takeuchi, Hideomi Yamashita, Keiichi Nakagawa

**Affiliations:** 1 Radiology, The University of Tokyo Hospital, Tokyo, JPN

**Keywords:** oligometastases, dwibs, bone metastasis, stereotactic body radiotherapy, castration-resistant prostate cancer

## Abstract

Diffusion-weighted whole-body imaging with background body signal suppression (DWIBS) is an emerging extended modality of diffusion-weighted imaging for screening malignant lesions in the body. A 70-year-old male was diagnosed with advanced-stage prostate adenocarcinoma with distant metastasis. After hormone therapy, the disease progressed to castration-resistant prostate cancer (CRPC). Serum prostate-specific antigen (PSA) levels increased during androgen deprivation therapy with low serum testosterone levels. 18F-Fluorodeoxyglucose positron emission tomography (FDG-PET) and technetium-99m methylene bone scintigraphy (BS) did not reveal obvious distant metastases; however, we were able to identify distant metastases by DWIBS. We herein report a case in which stereotactic body radiotherapy (SBRT) was performed on target lesions detected by DWIBS and successfully suppressed disease progression.

## Introduction

Discerning distant metastases is essential to determine recent cancer treatment strategies. Since the presence of distant metastases limits the efficacy of surgery and irradiation, it should always be located before initiating local treatment. Bone scintigraphy (BS) is often used for prostate cancer because of its tendency to metastasize to the bone. 18F-Fluorodeoxyglucose positron emission tomography (FDG-PET) is a powerful diagnostic imaging platform for the diagnosis of osseous and non-osseous metastases [1].

Diffusion-weighted whole-body imaging with background body signal suppression (DWIBS) is an emerging, extended modality of diffusion-weighted imaging for screening malignant lesions in the whole body. DWIBS was first reported by Takahara et al. in 2004 to detect systemic metastatic lesions throughout the body without irradiation [2]. We report a successful case of solitary bone metastasis detected by DWIBS, which was hardly confirmed by FDG-PET.

## Case presentation

**Figure 1:**
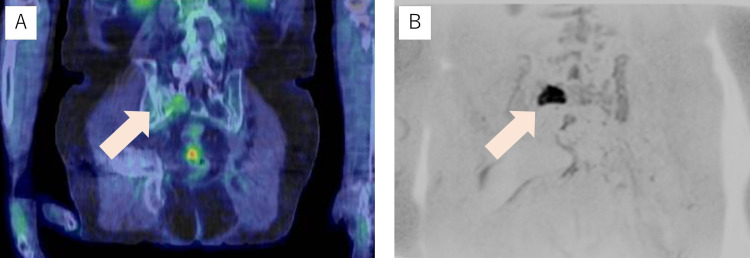
Coronal section image of the right sacral lesion (A) Coronal section image of the right sacral lesion in 18F-fluorodeoxyglucose positron emission tomography merged with computed tomography (arrow). (B) Coronal section image of the right sacral lesion in diffusion-weighted whole-body imaging with background body signal suppression (arrow).

A 70-year-old male was diagnosed with prostate adenocarcinoma with a Gleason score of 4+4 and a serum prostate-specific antigen (PSA) level of 63.44 ng/mL. He had a medical history of hepatitis C and duodenal ulcers treated surgically. Contrast-enhanced computed tomography (CT) revealed local invasion of the rectum and seminal vesicle, left internal iliac lymph node metastasis, and left femoral head bone metastasis. The clinical stage was T4N1M1b, stage IVB, according to the eighth edition of the TNM classification produced by the American Joint Committee on Cancer. He was started on androgen deprivation therapy, and his PSA level decreased to 0.24 ng/mL. However, the PSA serum level increased again to 3.27 ng/mL after 2.5 years from the initial diagnosis. Therefore, the patient was diagnosed with castration-resistant prostate cancer (CRPC). After 13 cycles of cytotoxic chemotherapy consisting of cisplatin and docetaxel, the PSA level decreased to 0.029 ng/mL; however, chemotherapy could not be continued owing to severe adverse events of dyspnea and right pleural effusion. He was then started on leuprolide with prednisone, but PSA gradually increased to 4.45 ng/mL. Technetium-99m methylene diphosphonate BS revealed no evidence of bone metastases. FDG-PET showed no evident distant metastasis except for slight fluorodeoxyglucose (FDG) accumulation at the standardized maximum uptake value (SUVmax) of 2.4 in the right sacral bone. This sacral lesion was not considered a metastasis from CRPC, and the patient decided to receive salvage stereotactic body radiotherapy (SBRT) to the primary prostate site, which was performed 6.5 years after the initial diagnosis. However, PSA still increased up to 11.8 ng/mL with suppression of serum testosterone < 0.04 ng/dL after five months from prostate bed SBRT. Where PET still exhibited weak FDG accumulation (SUVmax: 3.2), DWIBS clearly detected the right sacral bone, which demonstrated a high-signal intensity on diffusion-weighted imaging (Figure 1).

**Figure 2:**
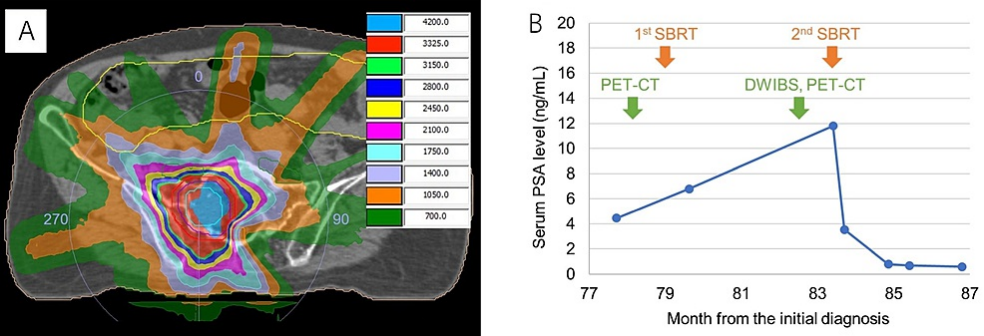
Radiation therapy planning and serum prostate-specific antigen trend (A) Radiation therapy planning for the right sacral lesion. The left table shows the radiation dose with a unit of centigray. (B) Serum prostate-specific antigen trend before and after stereotactic body radiotherapy (SBRT). The first and second SBRT was performed on the primary prostate site and on the right sacral lesion, respectively.

He was 77 years old at this time, and SBRT for solitary right sacral bone metastasis with 35 Gy in 5 Gy fractions was performed. Then, his PSA levels decreased to 0.60 ng/mL four months after SBRT (Figure 2).

## Discussion

The five-year survival rate for prostate cancer with distant metastasis is approximately 30%, which is extremely low compared to locoregional cases [3]. Prostate cancer responds well to hormone therapy, but several years after initial treatment, it becomes CRPC, which is a condition wherein the cancer is resistant to androgen deprivation therapy despite serum testosterone being as low as castration levels [4]. Tamada et al. reported that the median time to progression to CRPC in patients with prostate cancer who underwent hormone therapy was 40 months and even shorter (20 months) in high-risk patients [5]. Based on the presence of distant metastasis, CRPC can be classified as either nonmetastatic (m0CRPC) or metastatic (m1CRPC). Tombal insisted that m0CRPC and early m1CRPC have clearly different treatment endpoints from those of advanced m1CRPC [6]. In advanced m1CRPC, systemic therapy and palliative support are the mainstay of treatment. Contrarily, in m0CRPC and early m1CRPC, delaying the introduction of cytotoxic chemotherapy and stabilizing disease progression are important treatment endpoints. Our case was classified as early m1CRPC, and SBRT was successful in controlling the disease progression. SBRT against oligometastases may not only contribute to a local control effect but also enhance systemic T-cell antitumor immunity in patients with CRPC [7].

DWIBS is an emerging extended modality of diffusion-weighted imaging for screening malignant lesions [8]. There is no gold standard for detecting bone metastases in prostate cancer [9]. Bone metastasis is a condition that causes significant deterioration in the QOL, such as pathological fracture and spinal cord paralysis; thus, imaging diagnosis is necessary for early detection [10]. In recent years, it has become possible to detect metastases at the oligo-metastasis stage and treat them locally, leading to a better prognosis [11]. BS and FDG-PET-CT have been effective in the diagnosis of systemic bone metastases. Rodrigues et al. investigated the diagnostic performance of BS and FDG-PET-CT on the detection of bone metastases in patients with lung cancer [12]. According to their study, the sensitivity and specificity of FDG-PET-CT were 97.7% and 100%, respectively. Those of BS were 87.8% and 97.5%, respectively. Recently, technological advances have made it possible to perform a systemic search using DWIBS. Usuda et al. demonstrated that DWIBS has good accuracy for the characterization of recurrent metastatic lung cancer [13]. Their study revealed that DWIBS had a high accuracy of 0.98 for detecting metastatic lesions, which was compatible with that of PET-CT (accuracy: 0.94).

## Conclusions

In our case, DWIBS was useful for detecting solitary bone metastases, which were rarely detected by FDG-PET-CT and BS imaging. There is no uniform method for detecting bone metastases in prostate cancer. Unlike other imaging modalities that use radiation, DWIBS can be performed without being exposed to such. Evidence for the utilization of DWIBS is still accumulating; therefore, further clinical applications are expected in the future.
